# Cellular Basis of Organotin(IV) Derivatives as Anticancer Metallodrugs: A Review

**DOI:** 10.3389/fchem.2021.657599

**Published:** 2021-07-23

**Authors:** Sharifah Nadhira Syed Annuar, Nurul Farahana Kamaludin, Normah Awang, Kok Meng Chan

**Affiliations:** Center for Toxicology and Health Risk Studies, Faculty of Health Sciences, Universiti Kebangsaan Malaysia, Kuala Lumpur, Malaysia

**Keywords:** molecular, cytotoxicity, anticancer, application, ligand, organotin

## Abstract

Organotin(IV) compounds have wide applications in industrial and agricultural fields owing to their ability to act as poly(vinyl chloride) stabilizers and catalytic agents as well as their medicinal properties. Moreover, organotin(IV) compounds may have applications as antitumor, anti-inflammatory, antifungal, or antimicrobial agents based on the observation of synergistic effects following the binding of their respective ligands, resulting in the enhancement of their biological activities. In this review, we describe the antiproliferative activities of organotin(IV) compounds in various human cancer cell lines based on different types of ligands. We also discuss the molecular mechanisms through which organotin(IV) compounds induce cell death via apoptosis through the mitochondrial intrinsic pathway. Finally, we present the mechanisms of cell cycle arrest induced by organotin(IV) compounds. Our report provides a basis for studies of the antitumor activities of organotin(IV) compounds and highlights the potential applications of these compounds as anticancer metallodrugs with low toxicity and few side effects.

## Introduction

Tin is a silvery white metal often found in the form of tin dioxide (SnO_2_), derived from the Earth’s crust. The ability of tin to form stable bonds with carbon and other heteroatoms and its inert characteristics in the presence of air and water have attracted much interest in various research fields (Van der Kerk, 1975). Importantly, tin is present in a variety of inorganic/organometallic compounds. Its outer shell contains two 5s and two 5p electrons; loss of the two electrons in the 5p orbital or sharing of all four electrons with other atoms results in the formation of the Sn^2+^ ion and a stable electronic configuration. In aqueous media, the chemistry of tin is more complex; its stereochemistry involves the presence of inert electron pairs (resulting in stronger shielding of inner electrons compared with outer electrons). Sn^4+^ and Sn^2+^ ions, however, cannot be found in aqueous solutions. Organotin(IV) derivatives are formed via the binding of tin and carbon (Iqbal et al., 2015; Adeyemi and Onwudiwe, 2018). The molecular geometry of these compounds varies, including monomers, dimers, oligomeric ladders, cubics, butterflies, and hexameric drums, with the geometry playing a role in their biological activity (Holmes et al., 1988; Chandrasekhar et al., 2002; Chandrasekhar et al., 2005; Hadi et al., 2019a; Khan et al., 2020).

Organotin(IV) compounds also have their own role in commercial uses. According to Ross (1965) and Ghazi et al. (2018), organotin(IV) compounds are being utilized in three main areas, including 1) poly(vinyl chloride) (PVC) stabilizers, 2) industrial and agricultural biocides, and 3) catalytic agents. Since the 1940s, organotin(IV) compounds have been considered ideal stabilizer compounds to prevent thermal degradation of PVC (Martins et al., 2016). During processing, the required concentration for organotin(IV) compounds ranges from 0.5 to 3.0 parts per hundred parts of resin. The final product is a clear, colorless polymer because organotin(IV) compounds are compatible with PVC resins and plasticizers (Piver, 1973; Ghazi et al., 2018). Moreover, the capability of organotin(IV) compounds to control many fungi and bacteria enables organotin(IV)-based preservatives to be applied as industrial and agricultural biocides. In the 1960s, antifouling paint was applied in the production of marine vessels, rather than copper oxides; the organotin(IV) compounds, including tributyltin(IV) and triphenyltin(IV) compounds (e.g., oxide, chloride, acetate, and linoleate) were used to minimize fouling, leading to longer protection of up to 18–24 months (Omae, 2003). According to Piver (1973) and Sardon et al. (2013), polyurethane foams are typically prepared through a direct route involving hexamethylene diisocyanate and 1,4-butanediol with the presence of organotin(IV) catalysts. The main steps in this mechanism include chain extension, gas reaction, and crosslinking. The formation of urethane linkages involves either a Sn–O-isocyanate-insertion bond or the action of an organotin(IV) compound as a Lewis acid (Devendra et al., 2013). Besides, organotin(IV) compounds exhibit an interesting effect when catalyzing the reaction of chain extension, allowing optimum rates for both chain extension and gas formation to be achieved. The most commonly used organotin(IV) catalysts include dibutyltin(IV) diacetate, dibutyltin(IV) dilaurate, dibutyltin(IV) dichloride, dibutyltin(IV) dilaurylmercaptide, and dimethyltin(IV) dichloride (Piver, 1973; Ghazi et al., 2018).

Over 2000 tin-based pharmacological candidates have been tested by The National Cancer Institute (Nath and Saini, 2011). Organotin(IV) derivatives, hereby, have attracted much attention within the last 2 decades owing to their potential biological activities, including antitumor (Awang et al., 2011), anti-inflammatory (Niu et al., 2014), antimicrobial (Sedaghat et al., 2013), antifungal (Najeeb et al., 2009), antinematicidal, and anti-insecticidal effects (Jain et al., 2004). Driven by the clinical achievements of cisplatin, the first platinum-based chemotherapeutic drug, researchers have focused much attention on nonplatinum-based chemotherapeutics in order to improve therapeutic efficacy and prevent severe side effects related to platinum-based drugs. Among all reported nonplatinum-based drugs, organotin(IV) compounds may be the most promising metallodrugs, as, in some cases, they portray better effects than cisplatin (Hadjikakou and Hadjiliadis, 2009; Attanzio et al., 2020) and have been shown to have substantial anticancer effects (Jan et al., 2002; Samuel et al., 2002; Shuaibu et al., 2003; Tabassum and Pettinari, 2006).

Accordingly, in this review, we discuss the antiproliferative activities and mechanisms of organotin(IV) compounds to rationalize the potential of organotin(IV) compounds to be developed as effective and reliable anticancer agents in the future.

## Antiproliferative Activities of Organotin(IV) Compounds

Organotin(IV) compounds have attracted much attention recently as potential metallodrugs since the discovery of their antitumor activities (Gielen et al., 1999). Based on the wide range of organic moieties and donor ligands that are attached to the metal, several diorganotin(IV) and triorganotin(IV) compounds with *in vitro* antitumor properties against various solid and hematologic cancers have been studied (Gielen and Tiekink, 2005; Alama et al., 2009; Hadjikakou and Hadjiliadis, 2009). These compounds show lower toxic effects, higher antiproliferative activity, better excretion properties, and fewer side effects than other platinum-based drugs, even when used at low concentrations (Florea and Busselberg, 2011; Deo et al., 2018; Rubino et al., 2018; Attanzio et al., 2020). Additionally, novel organotin(IV) compounds have been shown to have high selectivity toward various cancer cell lines, regardless of ligand diversity (Awang et al., 2014; Girasolo et al., 2017; Attanzio et al., 2020). Niu et al. (2014), Sirajuddin et al. (2014) and Hadi et al. (2019a) stated that organotin(IV) derivatives are likely to show cytotoxic effects with the following trend: RSn^3+^ < R_2_Sn^2+^ < R_3_Sn^+^, with triorganotin(IV) substituents demonstrating the strongest effects. Table 1 shows the half-maximal inhibitory concentration (IC_50_) values for organotin(IV) derivatives against various human tumor cell lines. Here, we describe studies of the antiproliferative effects of these compounds and discuss their potential application in the treatment of cancer.

**TABLE 1:** Half-maximal inhibitory concentrations (IC_50_ values) of organotin(IV) derivatives.

No	Compounds	IC_50_ (μM)								References
		HeLa	A549	HCT-116	HepG2	MCF-7	K562	HL-60	Jurkat E6.1	
**1**	Et_2_SnL^1^	—	82.4	—	—	79.7	—	—	—	Devi et al. (2020)
**2**	Bu_2_SnL^1^	—	77.5	—	—	74.2	—	—	—	Devi et al. (2020)
**3**	Ph_2_SnL^1^	—	86.4	—	—	95.7	—	—	—	Devi et al. (2020)
**4**	Me_2_SnL^2^	—	94.1	—	—	91.2	—	—	—	Devi et al. (2020)
**5**	Et_2_SnL^2^	—	13.4	—	—	15.2	—	—	—	Devi et al. (2020)
**6**	Bu_2_SnL^2^	—	84.6	—	—	83.2	—	—	—	Devi et al. (2020)
**7**	Ph_2_SnL^2^	—	73.1	—	—	71.2	—	—	—	Devi et al. (2020)
**8**	Me_2_SnL^3^	—	85.7	—	—	82.5	—	—	—	Devi et al. (2020)
**9**	Et_2_SnL^3^	—	29.6	—	—	27.8	—	—	—	Devi et al. (2020)
**10**	Ph_2_SnL^3^	—	80.5	—	—	77.1	—	—	—	Devi et al. (2020)
**11**	Me_2_SnL^4^	—	107.9	—	—	103.2	—	—	—	Devi et al. (2020)
**12**	Et_2_SnL^4^	—	23.8	—	—	20.5	—	—	—	Devi et al. (2020)
**13**	Bu_2_SnL^4^	—	33.6	—	—	31.2	—	—	—	Devi et al. (2020)
**14**	Ph_2_SnL^4^	—	106.7	—	—	104.2	—	—	—	Devi et al. (2020)
**15**	n-Bu_3_Sn(5tpO)	—	—	0.07	0.07	0.23	—	—	—	Attanzio et al. (2020)
**16**	n-Bu_3_Sn(mtpO)	—	—	0.034	0.053	0.118	—	—	—	Attanzio et al. (2020)
**17**	n-Bu_3_Sn(HtpO_2_)	—	—	0.1	0.117	0.487	—	—	—	Attanzio et al. (2020)
**18**	Ph_3_Sn(HtpO_2_)	—	—	0.06	0.063	0.102	—	—	—	Attanzio et al. (2020)
**19**	(C_6_H_5_)_2_Sn[S_2_CN(C_3_H_7_O) (CH_3_)]_2_	—	—	—	—	—	4.0	—	—	Kamaludin et al. (2019)
**20**	(C_6_H_5_)_3_Sn[S_2_CN(C_3_H_7_O) (CH_3_)]	—	—	—	—	—	8.0	—	—	Kamaludin et al. (2019)
**21**	(C_4_H_9_)ClSnL_2_	758	—	—	—	—	—	—	—	Adeyemi et al. (2019)
**22**	(CH_3_)_2_SnL_2_	56	—	—	—	—	—	—	—	Adeyemi et al. (2019)
**23**	(C_4_H_9_)_2_SnL_2_	288	—	—	—	—	—	—	—	Adeyemi et al. (2019)
**24**	(C_6_H_5_)_2_SnL_2_	2	—	—	—	—	—	—	—	Adeyemi et al. (2019)
**25**	Me_2_SnL^1^	14.4	9.9	—	—	—	—	—	—	Liu et al. (2017)
**26**	Ph_2_SnL^1^	0.62	1.2	—	—	—	—	—	—	Liu et al. (2017)
**27**	Me_2_SnL^2^	34.69	19.50	—	—	—	—	—	—	Liu et al. (2017)
**28**	Ph_2_SnL^2^	1.25	3.4	—	—	—	—	—	—	Liu et al. (2017)
**29**	(C_4_H_9_)_2_SnL	—	—	—	—	—	—	0.40	—	Awang et al. (2016)
**30**	(C_6_H_5_)_2_SL	—	—	—	—	—	—	0.35	—	Awang et al. (2016)
**31**	n-Bu_3_SnCl	—	—	—	—	0.0027	—	—	—	Fickova et al. (2015)
**32**	Ph_3_SnCl	—	—	—	—	0.609	—	—	—	Fickova et al. (2015)
**33**	{[Ph_3_SnL]·0.5C_6_H_6_}_n_	1.76	—	—	—	—	—	—	—	Liang et al. (2014)
**34**	[Bu_2_LSnOSnLBu_2_]_2_	6.6	—	—	—	—	—	—	—	Liang et al. (2014)
**35**	Ph_2_Sn(mstsc)	—	—	—	—	—	—	—	0.3	Khandani et al. (2013)
**36**	Me_2_Sn(mstsc)	—	—	—	—	—	—	—	0.7	Khandani et al. (2013)
**37**	Bu_2_Sn(mstsc)	—	—	—	—	—	—	—	0.1	Khandani et al. (2013)

### Organotin(IV) Compounds Containing Hydrazone as Ligands

**SCHEME 1:**
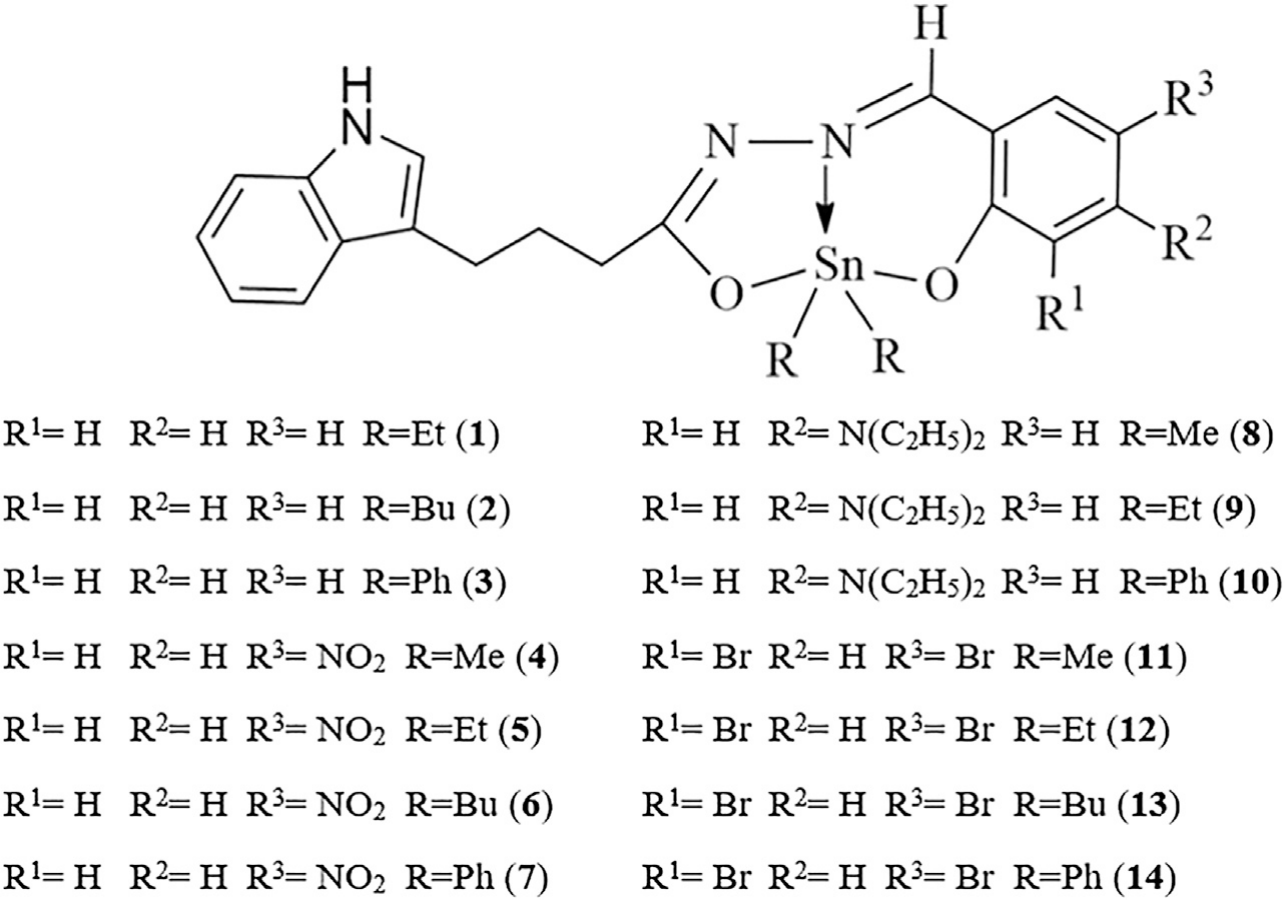
Chemical structure of **1–14**.

Devi et al. (2020) reported a series of diorganotin(IV) compounds derived from the reaction of indole-3-butyric hydrazide with salicylaldehyde and its derivatives (Scheme 1). The hydrazone Schiff base ligands were as follows: Nʹ-(2-hydroxybenzylidene)-4-(1H-indole-3-yl) butanehydrazide [H_2_L^1^], Nʹ-(2-hydroxy-5 nitrobenzylidene)-4-(1H-indole-3-yl)butanehydrazide [H_2_L^2^], Nʹ-(5-diethylamino)-2 hydroxybenzylidene-4-(1H-indole-3-yl)butanehydrazide [H_2_L^3^], and Nʹ-(3,5-dibromo-2 hydroxybenzylidene)-4-(1Hindole-3-yl)butanehydrazide [H_2_L^4^]. The geometry around the metal center indicates the tridentate nature of Schiff base ligands coordinated by nitrogen and oxygen donor sites to dialkyl/diaryltin(IV) moieties, yielding complexes with penta-coordinated geometry. The analysis of the cytotoxicity of the compounds in human lung epithelial (A549) and human breast adenocarcinoma (MCF7) cell lines showed that ethyl compounds displayed better cytotoxic activity toward both cancer cell lines. Compound **5** (Et_2_SnL2) exhibited the highest cytotoxic effects, with IC_50_ values of 13.4 μM (A549 cells) and 15.2 μM (MCF7 cells). This was followed by the ethyl compounds of H_2_L3 and H_2_L^4^ ligands, which resulted in stronger cytotoxic effects for both cell lines (IC_50_ values of less than 30 μM) in comparison with other compounds within the corresponding groups. Notably, when compared with the standard drug, doxorubicin, the ethyl compounds were approximately eight times less toxic in normal IMR90 cells. The antiproliferative activities of diorganotin(IV) compounds (**1–14**) decreased in the order of Et > Bu > Ph > Me.

**SCHEME 2:**
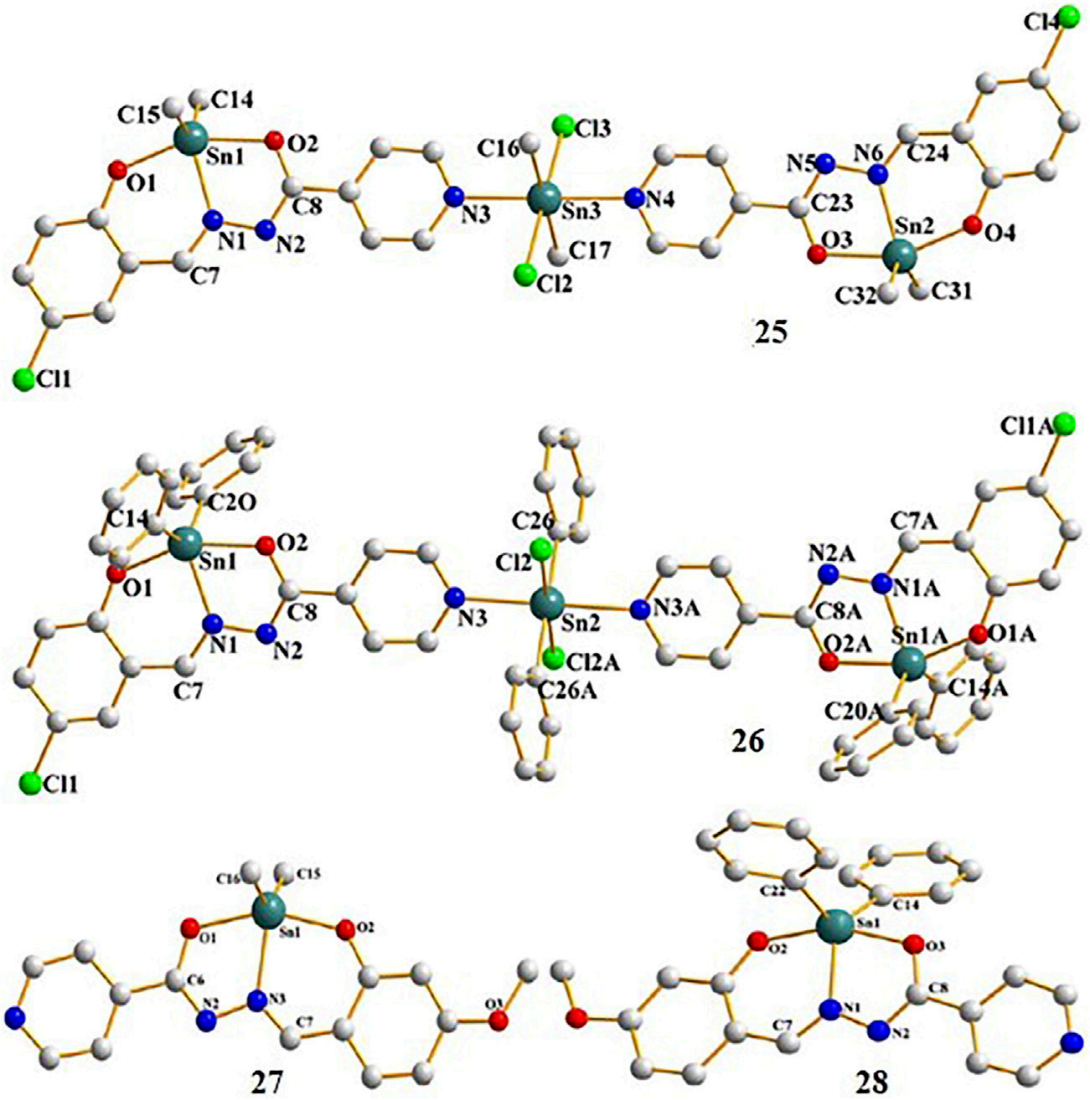
Chemical structure of **29–30**.

Liu et al. (2017) synthesized and characterized four new diorganotin(IV) compounds derived from two hydrazone Schiff base ligands (Scheme 2). The compounds were Me_2_SnL1 (**25**), Ph_2_SnL1 (**26**), Me_2_SnL2 (**27**), and Ph_2_SnL2 (**28**) (H_2_L1 = 5-chlorosalicylaldehyde isonicotinoyl hydrazone and H_2_L2 = 2-hydroxy-4-methoxybenzaldehyde isonicotinoyl hydrazone) with a trinuclear centrosymmetric structure (compounds **25** and **26**) or distorted trigonal bipyramid (compounds **27** and **28**) for the coordination environment. The *in vitro* cytotoxicity of the compounds was tested against A549 human lung cancer cells and HeLa human cervical carcinoma cells. The diphenyltin(IV) compounds **26** and **28** exhibited more potent cytotoxic activity than the dimethyltin(IV) compounds **25** and **27** in both cell lines. These results may be because the phenyl groups showed better planarity in compounds **26** and **28**, which may facilitate insertion into the DNA. These results were consistent with previous studies of organotin(IV) compounds conducted by Haque et al. (2015) and Shehata et al. (2015).

### Organotin(IV) Compounds Containing Dithiocarbamate as Ligands

**SCHEME 3:**
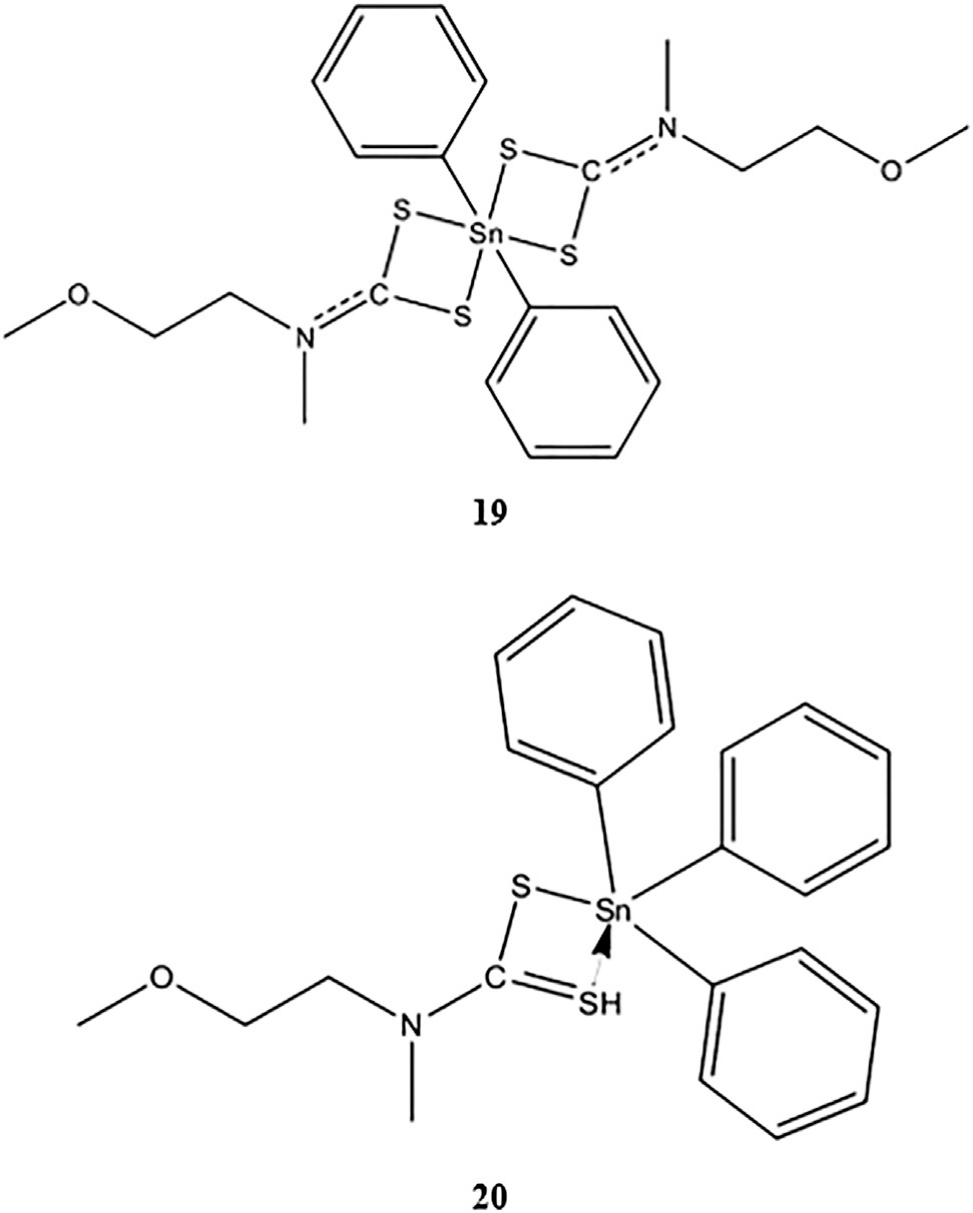
Chemical structure of **19**–**20** (Mohamad et al., 2016; Mohamad et al., 2018).

Phenyltin(IV) compounds containing (2-methoxyethyl) methyldithiocarbamate (Scheme 3) were examined to determine their *in vitro* cytotoxicity in K562 human erythroleukemia cells (Kamaludin et al., 2019). The cytotoxic activity of compound **19** was twice that of compound **20** (IC_50_ values 4.0 and 8.0 μM, respectively). Most studies have shown that triphenyltin(IV) compounds, which contain three phenyl groups attached to the central tin(IV) atom, have greater cytotoxic effects in tested cell lines compared with diphenyltin(IV) compounds. Additionally, Biplob et al. (2008) and Ray et al. (2000) demonstrated that the molecular structure of a compound plays important roles in determining its cytotoxicity, that is, shorter alkyl substitution groups were found to increase cytotoxicity.

**SCHEME 4:**
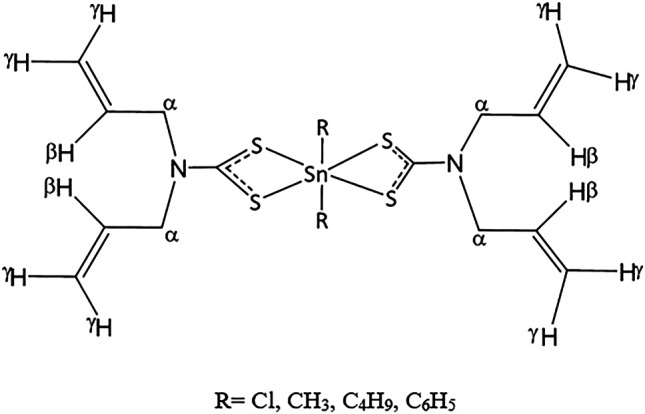
Chemical structure of **21–24**.

Four newly identified organotin(IV) compounds containing *N*,*N*-diallyldithiocarbamate (Scheme 4) were synthesized and characterized by Adeyemi et al. (2019). Compounds **22** and **23** were shown to have skewed trapezoidal–bipyramidal geometry. The antiproliferative activity of the compounds was evaluated in HeLa human cervical carcinoma cells, and the results showed that compound **24** possessed excellent cytotoxic effects (IC_50_ value as low as 2 μM). These findings may be explained by the lipophilicity of the compound, which was related to the presence of two phenyl groups (Kadu et al., 2015).

**SCHEME 5:**
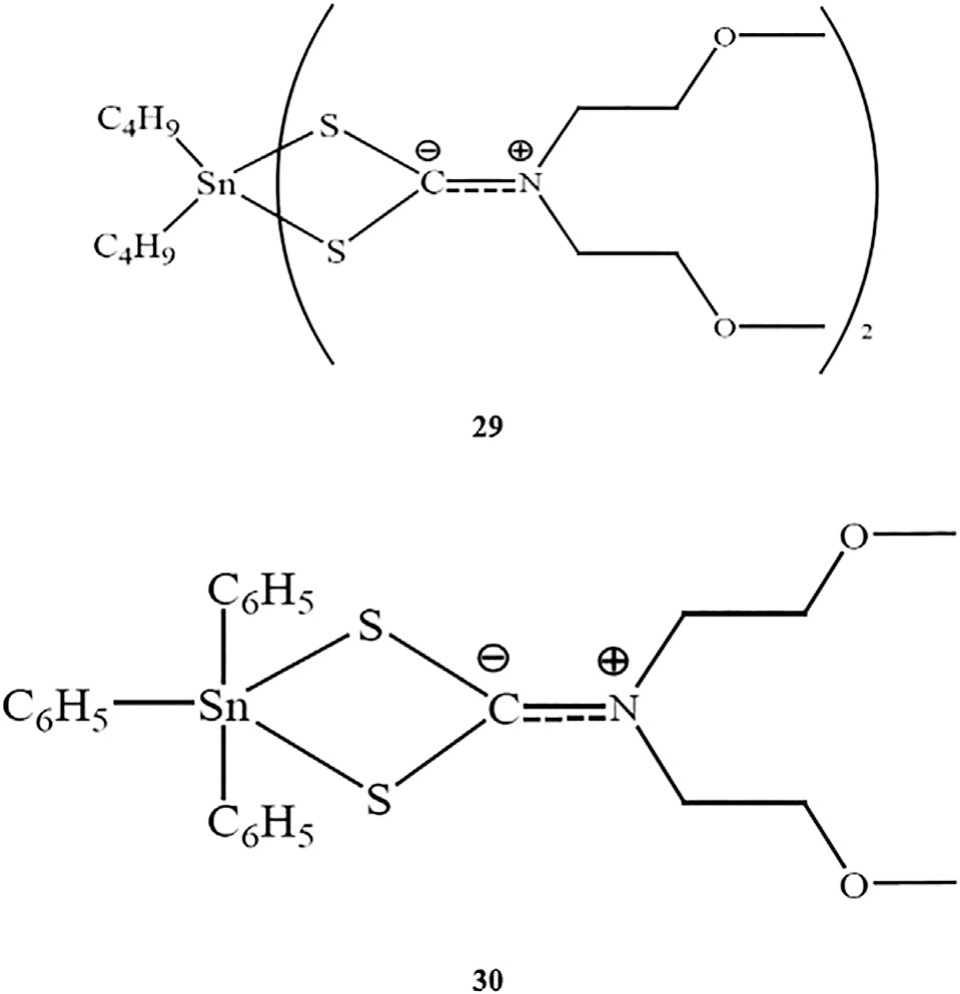
Chemical structures of the ligands and the atom numbering scheme.

Two novel organotin(IV) compounds as methoxyethyldithiocarbamate ligands (Scheme 5) were synthesized and characterized in a study by Awang et al. (2016). Further determination of the cytotoxic effects of dibutyltin(IV) and diphenyltin(IV) in HL-60 human leukemia cells showed that both compounds had very low IC_50_ values (0.40 and 0.35 μM for compounds **29** and **30**, respectively), supporting the strong cytotoxic activities of both compounds. These findings were consistent with a previous study by Awang et al. (2010) demonstrating the high cytotoxicity of dibutyltin(IV) compounds against tested cell lines.

Dithiocarbamate ligands have the potential to strongly bind and stabilize metal ions, resulting in a high oxidation number (Arafat et al., 2013). Chelation of the tin ion by dithiocarbamate ligands reduces the polar nature of the metal center, thereby enhancing permeability by increasing lipophilicity. This increased lipophilicity may enhance the biological activity of the compounds (Javed et al., 2016). Menezes et al. (2005) and Mamba et al. (2010) stated that the individual properties of organotin(IV) and dithiocarbamate constituents may induce synergistic effects, leading to better biological activities.

### Organotin(IV) Compounds Containing 1,2,4-Triazolo[1,5-a]Pyrimidines as Ligands

**SCHEME 6:**
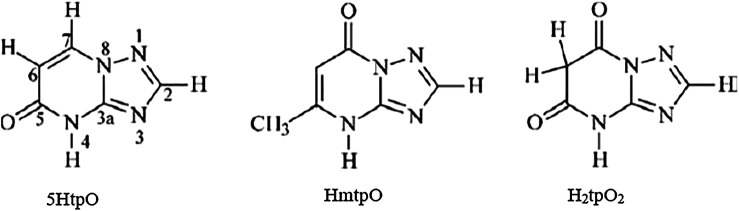
Molecular structure of **25–28** (Girasolo et al., 2005; Attanzio et al., 2020).

Attanzio et al. (2020) reported the *in vitro* antiproliferative activity of organotin(IV) compounds derived from 1,2,4-triazolo[1,5-a]pyrimidines (Scheme 6) in three different human tumor cell lines (HCT-116 colorectal carcinoma cells, HepG2 hepatocarcinoma cells, and MCF-7 breast cancer cells). The compounds were as follows: n-Bu_3_Sn(5tpO) (**15**), n-Bu_3_Sn(mtpO) (**16**), n-Bu_3_Sn(HtpO_2_) (**17**), and Ph_3_Sn(HtpO_2_) (**18**), where 5HtpO = 4,5-dihydro-5-oxo-[1,2,4]triazolo-[1,5-a]pyrimidine, HmtpO = 4,7-dihydro-5-methyl-7-oxo-[1,2,4]triazolo-[1,5-a]pyrimidine, and H_2_tpO_2_ = 4,5,6,7-tetrahydro-5,7-dioxo-[1,2,4]triazolo-[1,5 a]-pyrimidine. Interestingly, all of the compounds possessed IC_50_ values in the submicromolar range (less than 1 µM) against the three cell lines. Moreover, compound **16** showed the highest cytotoxic effects in all cell lines. All of the compounds showed high selectivity indexes (SIs) toward tumor cell lines (SI > 90).

The involvement of 1,2,4-triazolo[1,5-a] pyrimidine in the coordination of different nitrogen atoms could be used as a model system to mimic the reactivity between purines and several metal ions. Alterations in the numbers of exocyclic moieties on the triazolopyrimidine rings could increase the versatility and cytotoxicity properties of the compounds (Salas et al., 1999; Attanzio et al., 2020).

### Organotin(IV) Compounds Containing Chloride as Ligands

The antiproliferative effects of triorganotin(IV) compounds containing chloride (**31** and **32**) were assessed in MCF-7 human breast cancer cells. Fickova et al. (2015) showed that both tributyltin(IV) and triphenyltin(IV) had lower IC_50_ values (submicromolar range), demonstrating high potency of antitumor effects. Additionally, the tributyltin(IV) compound **31** showed greater growth inhibition in the tested cell lines compared with triorganotin(IV) (**32**). These findings were consistent with those reported by Attanzio et al. (2020) and Awang et al. (2016; 2011), who showed that butyltin(IV) compounds exhibited better cytotoxic effects than phenyltin(IV) compounds, regardless of the types of ligands attached to the central tin atom.

Organotin(IV) chloride derivatives are nanomolar agonists that can bind to retinoid X receptors (RXR) subtypes and peroxisome proliferator-activated receptor. These compounds also function as transcriptional activators. RXR subtypes modulate hormonal signals within the cells by acting as heterodimeric partners of various other nuclear receptors (Brtko and Thalhamer, 2003; Nakanishi et al., 2005; Brtko, 2007; le Maire et al., 2009; Brtko and Dvorak, 2011; Fickova et al., 2015). The organotin(IV) chloride compounds reported by Fickova et al. (2015) were shown to inhibit cell growth by modifying pro-apoptotic p53 and Bax and anti-apoptotic Bcl-2 protein levels in MCF-7 human estrogen receptor-positive breast adenocarcinoma cells; this signaling pathway was similar to that affected by other organotin(IV) derivatives.

### Organotin(IV) Compounds Containing (2E)-3-(3-Nitrophenyl)prop-2-Enoic Acid as Ligands

**SCHEME 7:**
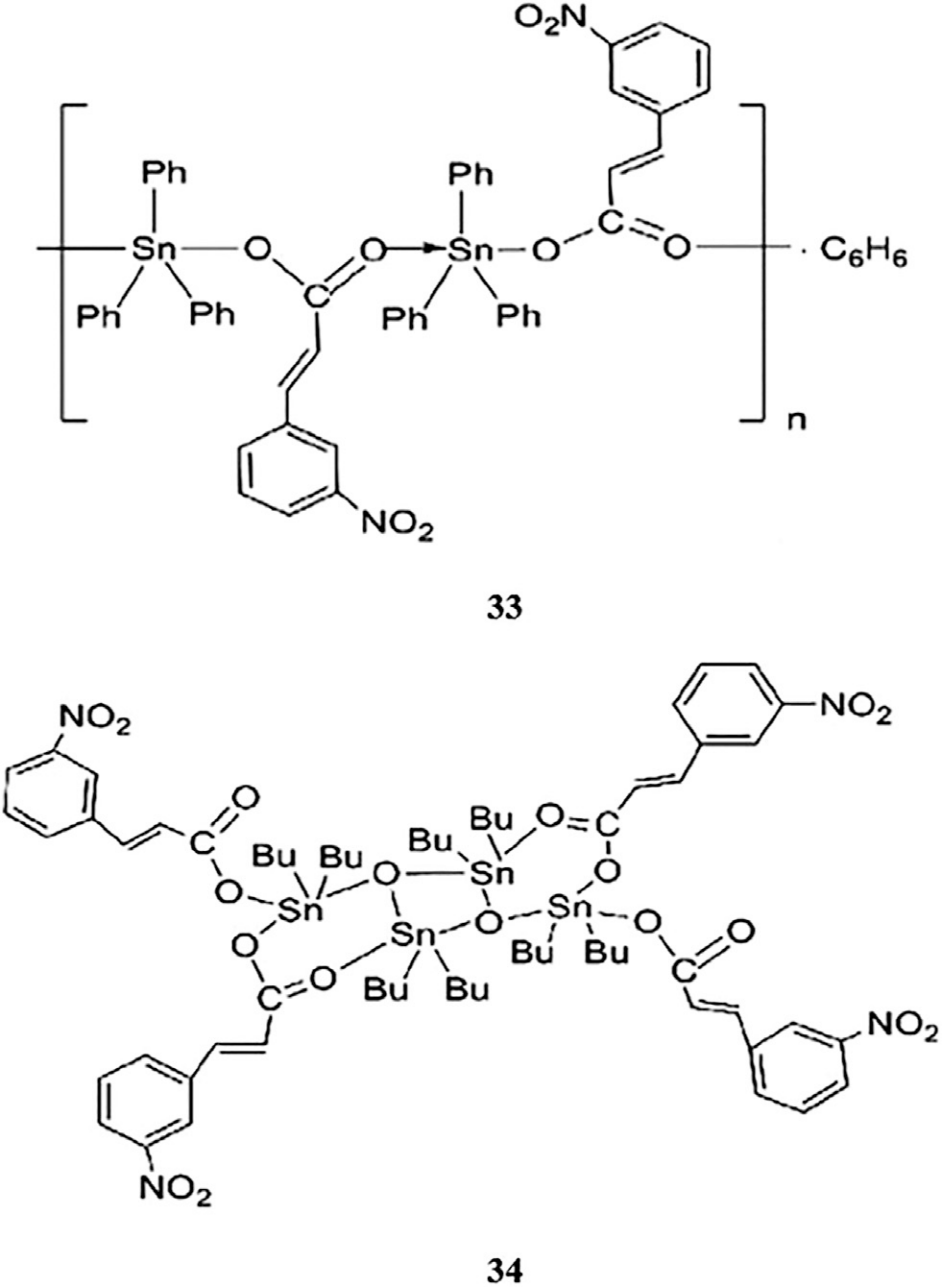
Chemical structure of **33–34**.

In a previous study, conducted by Liang et al. (2014), two novel organotin(IV) carboxylates of (2E)-3-(3-nitrophenyl)prop-2-enoic acid, compounds **33** and **34** (Scheme 7), showed concentration-dependent antitumor effects in HeLa cervical adenocarcinoma cells (IC_50_ values of 1.76 and 6.6 μM, respectively). The structures of the compounds included bridging of the carboxylate ligand of {[Ph_3_SnL]·0.5C_6_H_6_}_n_ (**33**), which generated one-dimensional polymeric chain structures around the five-coordinated tin centers. In contrast, [Bu_2_LSnOSnLBu_2_]_2_ (**34**) was characterized by a central rhombus Sn_2_O_2_ unit with two additional tin atoms linked at the O atoms. Overall, the findings showed that the triorganotin(IV) compound (**33**) showed higher antitumor activity than the diorganotin(IV) compound (**34**).

### Organotin(IV) Compounds Containing 3-Methoxysalicylaldehyde Thiosemicarbazone as Ligands

Compounds with 3-methoxysalicylaldehyde thiosemicarbazone (H_2_mstsc) were evaluated to determine their antitumor activity against Jurkat E6.1 acute lymphoblastic leukemia cells (Khandani et al., 2013). Three novel diorganotin(IV) compounds, namely Ph_2_Sn(mstsc) (**35**), Me_2_Sn(mstsc) (**36**), and Bu_2_Sn(mstsc) (**37**), were synthesized and characterized. Compound **36** and **37** adopted metal coordination geometry, including a distorted square pyramid, and the crystal lattices were found to be stabilized by intermolecular hydrogen bonds. The activities of the compounds decreased in the order of **37** > **35** > **36**; that is, the dibutyltin(IV) compound showed higher antitumor activity than the other two compounds. However, all compounds showed high toxicity, with IC_50_ values of less than 1 μM.

Overall, organotin(IV) constituents play a crucial part in inducing cytotoxicity, with the ligands being involved in transporting and addressing the molecule to the target while avoiding unwanted changes within the biomolecules (Alama et al., 2009). Many organotin(IV) derivatives were synthesized and characterized according to their respective ligands, including oxides, thiolates, carboxylates, halides, fluorides, and hydroxides (Gielen, 2003; Hadi et al., 2019b), some of which were mentioned previously (*Antiproliferative Activities of Organotin(IV) Compounds*). These compounds were then evaluated concerning their antiproliferative activity toward various cancer cell lines. The compounds with halides ligands (Fickova et al., 2015) and oxygen-donor ligands (Attanzio et al., 2020) revealed lower IC_50_ values, up to the submicromolar range. Moreover, most compounds can be classified as highly toxic since their IC_50_ values were lower than 7.34 μM, as mentioned by How et al. (2008). Noteworthy, the various antiproliferative activities of the compounds are mainly influenced by their structure. When the electron withdrawing group(s) are attached to the tin atom, they tend to reduce the electron density in the compound; thus, the changing in the alkyl or aryl substituent in an organotin(IV) compound may contribute to a significant effect on its biological activity (Pellerito and Nagy, 2002; Iqbal et al., 2016). Moreover, the three main factors that are the nature of the organic R group, of the halide or pseudohalide X and of the donor ligand L, play a vital role in the structure and activity of organotin(IV) derivatives (L)_x_R_n_SnX_4-n_ (Hadi et al., 2019b). In addition, the number of the alkyl substituent and its length in the organotin(IV) moiety may also contribute to the desirable cytotoxicity on various cell lines, among which the toxicity will become lesser with longer alkyl chains (Van der Kerk and Luijten, 1954; Van der Kerk and Luijten, 1956; Kamaludin et al., 2013; Hadi et al., 2019a). For example, the enzyme inhibition activity of the five-coordinated organotin(IV) carboxylates is higher than that of six-coordinated compounds (Hadi et al., 2019a), suggesting that the activity of the organotin(IV) compound can be effectively modulated by selected ligand, thereby potentially overcoming the compound limitations (Pellerito et al., 2006).

## Molecular Mechanisms Underlying Organotin(IV) Compounds

The antitumor activities of organotin(IV) compounds have been extensively studied (Shaheen et al., 2017), and most previous studies have been primarily focused on synthesizing, characterizing, and screening the antitumor activities of these organotin(IV) compounds. However, the mechanism of toxicity has not been clarified for this class of metal-based drugs, particularly in *in vivo* studies. Therefore, information on the mechanism of action has mainly been obtained in studies of cancer cell lines. For example, Barbieri et al. (2001) showed that tin(IV) compounds induce cell death via stimulation of apoptosis, thereby resulting in antitumor toxicity. Consistent with this, we have found that most organotin(IV) compounds induce cell death via apoptosis, specifically the intrinsic mitochondrial pathway, regardless of the donor-ligand.

**FIGURE 1:**
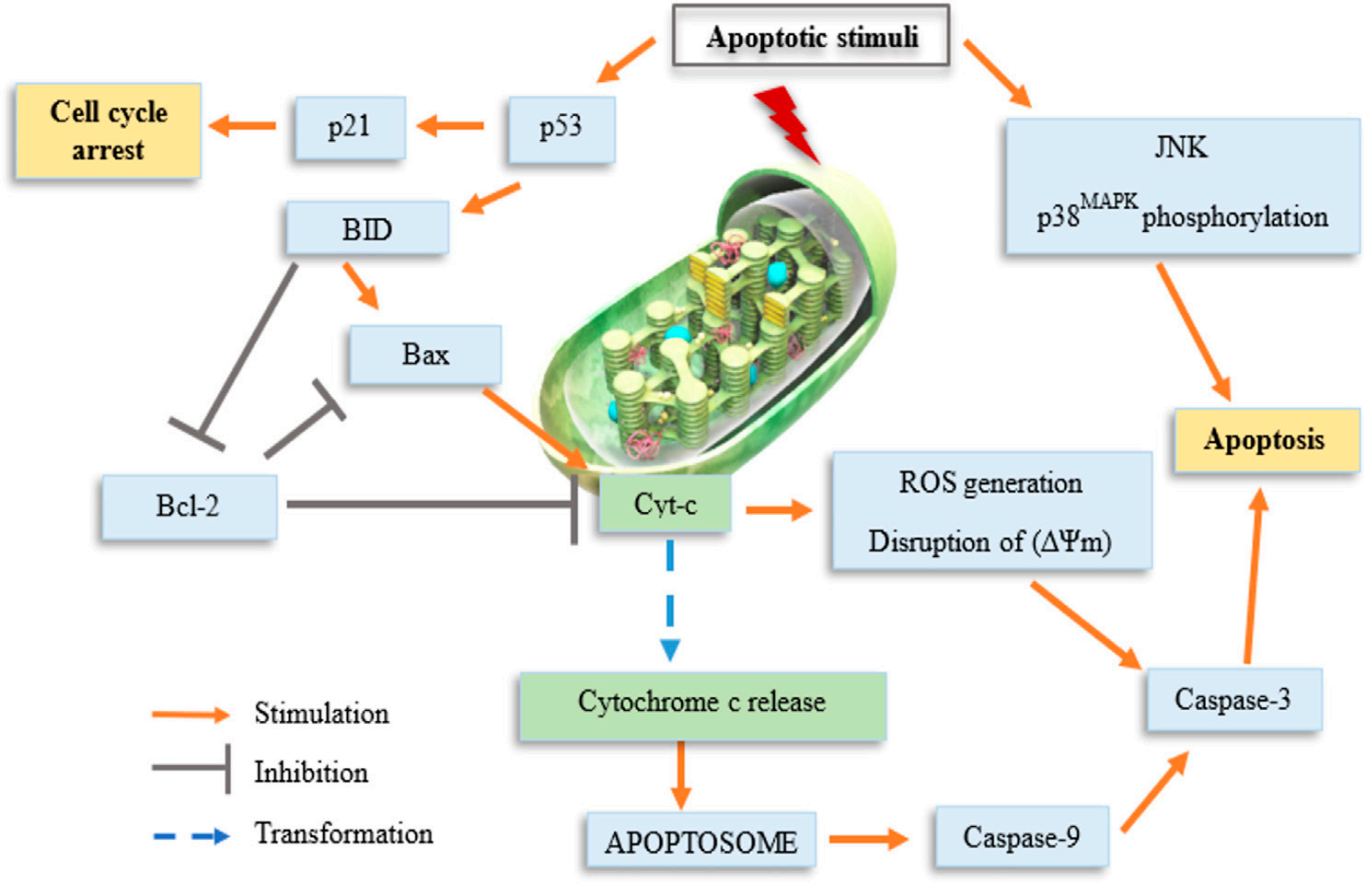
Schematic representation of organotin(IV) compounds-induced cell cycle arrest.

Some previous researchers have evaluated the molecular mechanisms underlying the cytotoxicity of organotin(IV) compounds, demonstrating the involvement of cell death via apoptosis (Ge et al., 2013; Girasolo et al., 2017; Kamaludin et al., 2019; Attanzio et al., 2020), and cell cycle arrest (Yunlan et al., 2014; Asanagi et al., 2016; Attanzio et al., 2020). Organotin compounds were said to induce apoptotic cell death by binding to DNA at external phosphate groups subsequently, altering phospholipid intracellular metabolism (Attanzio et al., 2020). Figure 1 shows a schematic representation of the potential mechanisms of organotin(IV)-induced apoptosis and cell cycle arrest in cancer cells. Moreover, we showed that the mechanisms of organotin(IV) compounds (regardless of the type of their donor-ligand) are similar to those of cisplatin, suggesting that organotin(IV) compounds may have potential applications as metal-based anticancer drugs.

The two main cell death mechanisms are apoptosis and necrosis; which mechanism is induced depends on the specific stimulus encountered and the amount of cellular energy available (Bjelaković et al., 2005). Cell death via apoptosis requires more energy via necrosis and involves the cleavage of cellular proteolytic substrates during intermediate stages. Activation of cysteine-aspartic proteases (caspases) leads to cell death via induction of DNA breaks and disruption of the adhesion of cytoskeletal proteins. Moreover, in apoptosis, there is no occurrence of enzyme release into the extracellular environment, thereby preventing damage to neighboring cells and avoiding stimulation of the immune response (Savill, 1997). Necrosis is a type of uncontrolled cell death typically triggered by external damage, such as hypoxia and inflammation. Hence, the cytoplasmic contents of the cell may be released into the surrounding tissues, resulting in widespread damage (Elmore, 2007; D’Arcy, 2019). Table 2 shows the differences between apoptosis and necrosis (Kerr et al., 1972; Majno and Joris, 1995; Trump et al., 1997; Elmore, 2007; Panawala, 2017; D’Arcy, 2019). In apoptosis, inflammation may not occur owing to lack of release of cellular contents into the surrounding microenvironment, the immediate phagocytosis of apoptotic cells prior to the formation of secondary necrosis, and lack of dissemination of anti-inflammatory cytokines by engulfing cells (Savill and Fadok, 2000; Kurosaka et al., 2003).

**TABLE 2:** Differences between apoptosis and necrosis.

Characteristics	Apoptosis	Necrosis
Type of cell death	Controlled cell death	Uncontrolled cell death
Cytoplasmic contents	Contents of the cells are not released into the surrounding environment for engulfment by macrophages	Release of cellular contents into the surrounding environment
Process	Apoptosis initiation depends on the activation of a sequence of caspases	Involves numerous pro-inflammatory factors, including proteins such as nuclear factor ĸB, leading to disruption of the cell membrane
Morphology	1. Cell shrinkage	1. Cell swelling
	2. Condensation of chromatin	2. Formation of cytoplasmic vacuoles
	3. Formation of apoptotic bodies	3. Detachment of ribosomes
	4. No occurrence of inflammation	4. Condensation; swelling and rupture of mitochondria, lysosomes, and organelle membranes
		5. Inflammation

**FIGURE 2:**
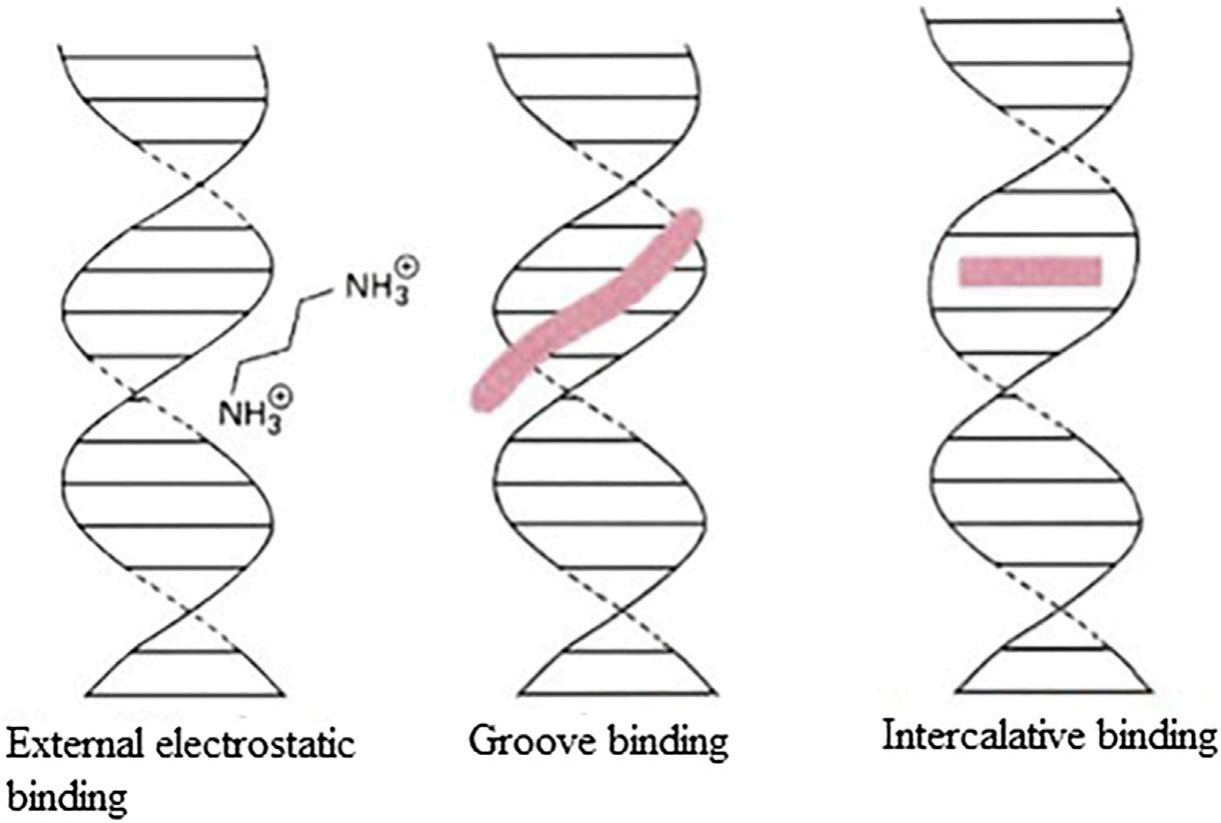
Three types of reversible DNA binders.

Hereby, the mechanism of action of organotin(IV) compound may involve triggering of the intrinsic apoptosis pathway, leading to the generation of DNA damage, oxidative stress, and subsequently, the activation of p53 (Ge et al., 2013; Fickova et al., 2015; Girasolo et al., 2017; Attanzio et al., 2020). Most cytotoxic drugs target DNA (Tabassum and Pettinari, 2006; Yusof et al., 2019). Figure 2 shows three types of reversible DNA binders (Silverman and Holladay, 2015). Previous mechanistic studies have shown that organotin(IV) compounds can interact with DNA as intercalative binding-type or groove binding-type drugs depending on the coordination number of the attachment and the nature of the groups attached to the central tin atom (Tabassum and Pettinari 2006; Rehman et al., 2016; Sirajuddin et al., 2016; Liu et al., 2017; Yusof et al., 2019). The ability of these compounds to interact with DNA (binding or cleaving) is thought to arise from the biological potency of the compounds (Yusof et al., 2019).

Oxidative stress can also be described as the disruption of the pro-oxidant/antioxidant balance in cells, resulting in substantial oxidative damage and reduced cell viability (Sinha et al., 2013). Accumulation of reactive oxygen species (ROS) following induction of oxidative stress results in oxidative damage to essential biomolecules, such as proteins, lipids, and nucleic acids (Bilinski et al., 1989; Yakes and VanHouten, 1997; Cabiscol et al., 2000; Sinha et al., 2013). The three main types of ROS include hydrogen peroxide (H_2_O_2_), hydroxyl radical (·OH), and superoxide anion (O_2_·-) (Mate’s et al., 2012). The formation of H_2_O_2_ by O_2_·- or by the action of oxidase enzymes can lead to damage. Moreover, leakage in the mitochondrial respiratory chain may cause the emergence of O_2_·-. The most damaging type of ROS is ·OH, which can alter DNA bases and induce DNA strand breaks, eventually resulting in DNA damage (Mate’s et al., 2010; Mate’s et al., 2012). Mitochondria are the sites of ROS accumulation (mainly O_2_· and ·OH) because most ROS generation occurs in the mitochondria. This may cause deleterious effects, including oxidative impairment of mitochondrial DNA and subsequent cell death via apoptosis (Orrenius et al., 2007; Ricci et al., 2008; Circu et al., 2009; Rachek et al., 2009; Sinha et al., 2013) as demonstrated by a treatment with organotin(IV) compounds.

The organotin(IV) compounds were reported to cause disruption in mitochondrial membrane following the activation of p53 that induces BH3 pro-apoptotic proteins, such as BID and BIM, which are translocated to the mitochondria (Riedl and Shi, 2004; Attanzio et al., 2020). The molecular mechanism that involves in the activation of p53 resulted in targeted cytotoxic effects via apoptosis or cell cycle arrest. p53 is a major tumor-suppressor gene that regulates various biological activities, including cell cycle arrest, cellular aging, DNA repair, and apoptosis (Kastenhuber and Lowe, 2017). Additionally, via its function as a transcription factor, p53 forms homotetramers that can activate almost 500 target genes and regulate apoptosis signaling (Riley et al., 2008; Aubrey et al., 2018).

The loss in mitochondrial membrane of the cancer cells induced by organotin(IV) compounds causing the release of mitochondrial cytochrome c into the cytosol that is regulated by Bcl-2 family proteins (e.g., Bax and Bak). This facilitates the formation of the apoptosome complex (composed of cytochrome c, the apoptotic molecule activating factor protease 1, and pro-caspase-9). This complex then induces caspase-9 activation via cleavage, followed by activation of the effector caspase, caspase-3, which executes the final steps of apoptosis (Green and Kroemer, 2004; Mohamad et al., 2005; Er et al., 2006; Giam et al., 2008; Ge et al., 2013). Caspases are defined as synthesized as zymogens and are then converted from precursors to mature proteases. Initiator caspases are activated by the oligomerization autoprocess; effector caspases are activated by other proteases, including the initiator caspase and granzyme B (Li et al., 1997; Muzio et al., 1997; Butt et al., 1998; Martin et al., 1998; Srinivasula et al., 1998; Yang et al., 1998). In addition, there are two types of overlapping signaling pathways, that is, the intrinsic and extrinsic pathways, which promote apoptosis. The type of pathway activated depends on the expression of specific initiator caspases. The intrinsic pathway is generally activated by mitochondrial dysfunction, oxidative stress, and pro-apoptotic factor expression, whereas the extrinsic pathway is activated by ligand attachment on cell death receptors (Bai and Odin, 2003). Hence, this explained that organotin(IV) compounds indeed induce apoptotic cell death via intrinsic mitochondrial pathway. Apart from that, according to Ge et al. (2013), the mitogen-activated protein kinases JNK and p38MAPK are also responsive to various types of stress stimuli and participate in the induction of apoptosis by organotin(IV) compounds via modulation of phosphorylation.

In addition to modulation of apoptosis, cancer cells also exhibit loss of cell cycle control, resulting in dysregulated cell division and proliferation (Senese et al., 2014; Kartal-Yandim et al., 2015) and lack of cell cycle arrest upon induction of cellular stress. The cell cycle checkpoint can prevent DNA damage in cells in response to chemotherapeutic drugs (Sancar et al., 2004), giving the cells a sufficient amount of time to recover. In contrast, carcinogenesis can cause disruption of all cell cycle checkpoints. However, Linke et al. (1996) showed that treatment with chemotherapeutic drugs and gamma radiation may trigger DNA damage, resulting in cell cycle arrest at G0/G1 phase. This is aligned with the previous studies that described the involvement of the p53/p21WAF1 signaling pathway in cell cycle arrest induced by organotin(IV) compounds.

**FIGURE 3:**
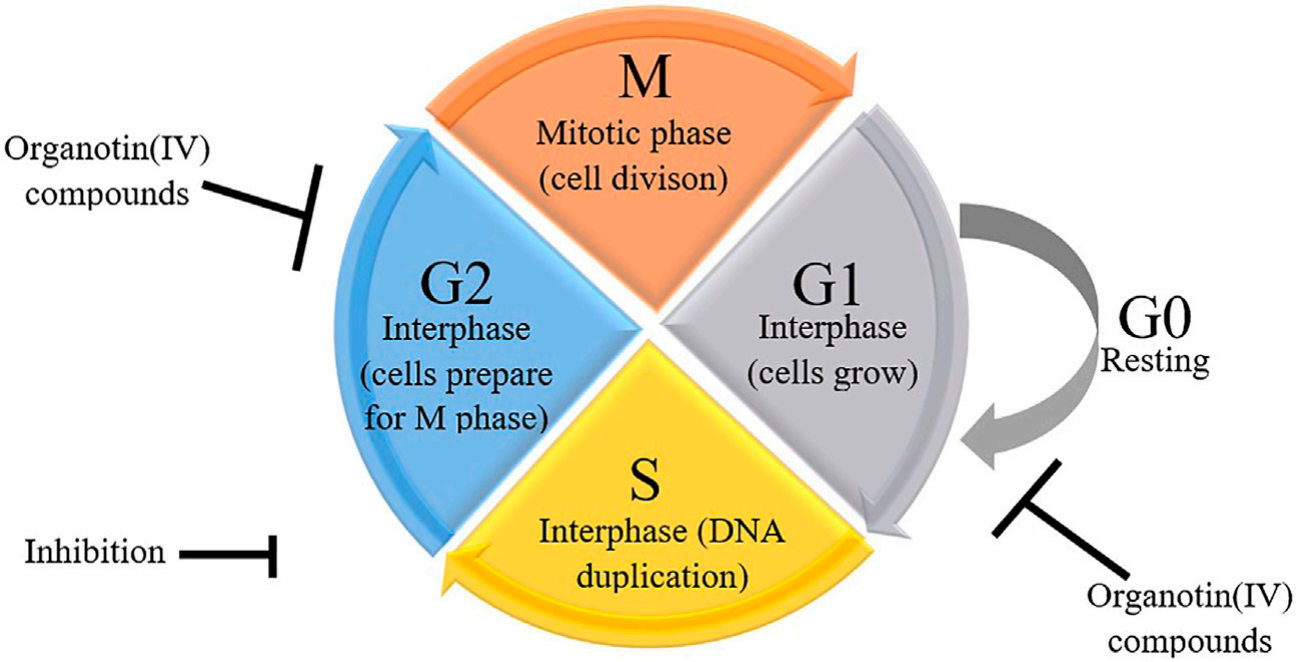
Schematic representation of possible mechanisms of organotin(IV) compounds.

The occurrence of cell cycle arrest at different phases is modulated by p53-dependent transcriptional activation of p21WAF1, which binds to and regulates several cyclins (Abbas and Dutta, 2009; Chen, 2016). During G1 phase, cells enter the cell cycle and prepare themselves for DNA duplication in S phase. Subsequently, in G2 phase, DNA damage is repaired before cells enter M (mitosis) phase, during which chromatids and daughter cells separate (Bible and Kaufmann, 1997; Carlson et al., 1999; Jackson et al., 2000; Senderowicz and Sausville, 2000; Hirose et al., 2001; Sausville et al., 2001; Shapiro et al., 2001). The cells then enter a quiescent (inactive) stage. Disruption of G2 phase may allow the cells to preserve their viability following induction by drugs that affect the main checkpoint kinase, Chk1 (Hapke et al., 2001; Xiao et al., 2003). Indeed, cell cycle checkpoints are considered strong drug targets because of their ability to control the cell cycle. Hence, researchers in the field of anticancer drugs often evaluate the effects of drugs on the cell cycle in cancer cells. Organotin(IV) compounds have been shown to cause G2/M phase arrest in several cancer cell lines (Yunlan et al., 2014; Asanagi et al., 2016; Attanzio et al., 2020) and G0/G1 phase arrest in other cancer cell lines (Attanzio et al., 2020) as shown in Figure 3. The arrest of proliferation at different cell cycle phases highlights the important properties of organotin(IV) compounds and ligand moieties in modulating biological activity.

As described above, most studies of organotin(IV) compounds have been performed in cancer cell lines. However, Verginadis et al. (2011) and Barbieri et al. (2000) found that organo-based compounds could also show antitumor activity in tumor-bearing animals. Indeed, triorganotin(IV) compounds show potent antitumor activities and prolong survival time in tumor-bearing animals (Barbieri et al., 2000).

## Concluding Remarks

In this review, we discussed the strong cytotoxic effects of organotin(IV) compounds. Most organotin(IV) compounds have been shown to activate apoptosis via the intrinsic mitochondrial apoptosis pathway, suggesting potential applications in anticancer chemotherapy (Cima and Ballarin, 1999; Pellerito et al., 2005). The ideal characteristics of anticancer drugs include the ability to induce apoptotic cell death (Yamaguchi et al., 2007; Husaini et al., 2017) and selectivity toward cancerous cells. Thus, organotin(IV) compounds show high potential for use as chemotherapeutic metallopharmaceuticals based on their excellent cytotoxic characteristics. However, more studies of the mechanisms of action of these compounds both *in vitro* and *in vivo* are required in order to elucidate the exact mechanisms through which cell death is induced.
